# Transplantation to study satellite cell heterogeneity in skeletal muscle

**DOI:** 10.3389/fcell.2022.902225

**Published:** 2022-08-24

**Authors:** Bahareh Hekmatnejad, Michael A. Rudnicki

**Affiliations:** ^1^ The Sprott Centre for Stem Cell Research, Regenerative Medicine Program, Ottawa Hospital Research Institute, Ottawa, ON, Canada; ^2^ Department of Cellular and Molecular Medicine, Faculty of Medicine, University of Ottawa, Ottawa, ON, Canada

**Keywords:** satellite cells, muscle stem cell, self-renewal, differentiation, engraftment, transplantation, heterogeneity

## Abstract

Skeletal muscle has a remarkable capacity to regenerate throughout life, which is mediated by its resident muscle stem cells, also called satellite cells. Satellite cells, located periphery to the muscle fibers and underneath the basal lamina, are an indispensable cellular source for muscle regeneration. Satellite cell transplantation into regenerating muscle contributes robustly to muscle repair, thereby indicating that satellite cells indeed function as adult muscle stem cells. Moreover, satellite cells are a heterogenous population in adult tissue, with subpopulations that can be distinguished based on gene expression, cell-cycle progression, ability to self-renew, and bi-potential ability. Transplantation assays provide a powerful tool to better understand satellite cell function *in vivo* enabling the separation of functionally distinct satellite cell subpopulations. In this review, we focus on transplantation strategies to explore satellite cells’ functional heterogeneity, approaches targeting the recipient tissue to improve transplantation efficiency, and common strategies to monitor the behaviour of the transplanted cells. Lastly, we discuss some recent approaches to overcome challenges to enhance the transplantation potential of muscle stem cells.

## Introduction

Skeletal muscle exhibits a strong capability for tissue expansion and repair. It is a striated muscle tissue, which accounts for a large portion of adult human body weight. Skeletal muscle consists of highly specialized post-mitotic, large multinucleated cells referred to as myofibers, which can contain thousands of nuclei, that form following fusion from myogenic progenitors. During postnatal growth, the number of myofibers does not increase in number, but each myofiber grows in size by fusion of muscle stem cells (MuSCs), also known as satellite cells, readily adapting to changing functional needs.

Satellite cells were described in 1961 by Alexander Mauro as mononucleated cells “wedged” during the characterization of frog myofibers by electron microscopy (Mauro, 1961). Satellite cells are located between the basal lamina and sarcolemma of the muscle fiber. Under normal physiological conditions, they are actively maintained in a quiescent state in adult muscle and are identified through the expression of the transcription factor Pax7 (Seale et al., 2000). In response to physiological insults, such as an injury or exercise, they quickly become activated, enter the cell cycle, and give rise to proliferating myoblasts that eventually differentiate and fuse to repair damaged myofibers (Bentzinger et al., 2012). A subset of activated MuSCs, are also able to undergo self-renewal to maintain the muscle stem cell reservoir for future regeneration. Therefore, the interplay between extrinsic and intrinsic mechanisms tightly controls the balance between the satellite cells committed to forming new myofibers and those to self-renew (Kuang et al., 2007; Rocheteau et al., 2012a).

Transplantation studies involving satellite cells derived from adult muscles allow researchers to explore satellite cell functional heterogeneity (including self-renewal, differentiation, and multipotential specification). Transplantation of skeletal muscle came into play in the late 1980s by the work of Partridge’s group (Partridge et al., 1989) who demonstrated that injecting myoblasts into the hind limb of the *mdx* mouse model for DMD (Duchenne muscular dystrophy) resulted in a significant reconstitution of dystrophin positive muscle fibers. These initial studies on myoblast transplantation resulted in low survival rates post-injection with a minimal number of donor cells exhibiting stem cell properties that behave as long-term repopulating cells and contribute to muscle homeostasis (Beauchamp et al., 1999). Typically, transplantation experiments involve the isolation of cells from dissected muscle by mechanical and enzymatic digestion. Muscle stem cells are then enriched by FACS (fluorescent activated cell sorting), using MuSC-specific cell surface markers (α7-integrin, Vcam1, and CD34) and negative markers (CD31, CD45, CD11b, and Sca1) (Pasut et al., 2012; Maesner et al., 2016). Lastly, the isolated muscle stem cells are injected into a recipient muscle tissue.

Lineage tracing together with engraftment studies in mice have identified a putative long-term self-renewing stem cell within the satellite cell population (Kuang et al., 2007; Wang et al., 2019). Muscle stem cells undergo planar-symmetric divisions to give rise to two stem cells. Alternatively, they undergo an apical-basal asymmetric division to give rise to a stem cell and a committed cell (Kuang et al., 2007; Wang et al., 2019; Feige and Rudnicki, 2020). Thus, the majority of satellite cells represent a short-term repopulating cell (Kuang et al., 2007; Feige et al., 2018), while a subset we term muscle stem cells can self-renew over a long period of time and can rise to committed progenitors via asymmetric cell divisions (Kuang et al., 2007; Rocheteau et al., 2012b; Gurevich et al., 2016; Wang et al., 2019; Evano et al., 2020).

In this review, we first summarize studies utilizing transplantation experiments to measure satellite cell functional capabilities. We then discuss evidence indicating that satellite cells are indeed the repopulating cell. Moreover, we review different preconditioning strategies of host tissue to enhance engraftment efficiency in animal models, and common methods to monitor the engraftment of host cells. We note that poor engraftment is one of the greatest challenges limiting the success of cell transplantation experiments, which depends on several parameters such as survival, host immune response, migration to the degenerating tissue, and differentiation of the transplanted cells. Therefore, we provide discussion with possible approaches to overcome the biological challenges associated with engraftment that could be applied to help improve the outcome.

## Satellite cells represent the ideal source for muscle transplantation

Satellite cells are widely recognized as the most crucial stem cell type for muscle repair with tremendous *in vivo* regenerative potential. Transplantation of a single fiber or even a single satellite cell contributes considerably to the regeneration of the damaged skeletal muscles by participating in the reconstitution of both the fiber and the muscle stem cell niche (Collins et al., 2005; Sacco et al., 2008).

Over the past decade, studies using single-cell technologies together with engraftment experiments demonstrated that satellite cells are functionally and molecularly heterogenous. Our group and other researchers have characterized subpopulations of Pax7-expressing satellite cells with varying functional potential (Kuang et al., 2007; Sacco et al., 2008; Rocheteau et al., 2012a; Chakkalakal et al., 2014; Scaramozza et al., 2019). We identified a small subset of the satellite cells that have increased levels of Pax7 and lack Myf5 (Pax7^+^/Myf5^−^), demonstrating a greater ability for self-renewal compared with their more committed progenitors (Pax7^+^/Myf5^+^) (Kuang et al., 2007). Chakkalakal and colleagues (Chakkalakal et al., 2012; Chakkalakal et al., 2014) used a doxycycline (DOX)-inducible TetO-H2B-GFP reporter system to examine the satellite cell heterogeneity with regard to their cell cycle dynamics. Mice containing the Histone2B (H2B)-Green Fluorescent Protein (GFP) fusion protein were transiently subjected to doxycycline to achieve widespread incorporation of the fusion protein into chromatin. Following doxycycline withdrawal, mice were chased for the retention of H2B-GFP label at defined intervals. The GFP label is retained in quiescent non-dividing cells, while it is diluted by half with each cell division in dividing cells (Foudi et al., 2009). The authors demonstrated that the adult satellite cell pool is composed of ∼30% label-retaining satellite cells (LRCs), while the vast majority lost it (non-LRCs). They showed that both these cell populations are present at birth and persist throughout postnatal development and adult life. Furthermore, transplantation studies showed that LRCs function similar to stem cells, generating self-renewing cells capable of differentiation. Meanwhile, non-LRCs are restricted to differentiation, thereby functioning as committed progenitors (Chakkalakal et al., 2012; Chakkalakal et al., 2014). More recently, employing *Mx1-Cre* transgenic reporter mice, which allows monitoring of resident stem cells in the majority of adult tissues, Scaramozza et al. (2019) identified a rare subset of Pax7^+^ satellite cells that are enriched for both the *Mx1-Cre* and *Pax3* expression. After irradiation, the Mx1^+^ SCs undergo clonal expansion and contribute extensively to muscle repair and niche repopulation. Therefore, the radiotolerant Pax7^+^ Mx1^+^ subpopulation functions as a reserve muscle stem cell population. Of note, examination of the lineage commitment using markers of self-renewal and differentiation, suggested that Mx1^+^ satellite cells and LRCs display cellular and functional overlap, and thus they have similar fate biases.

Notably, apart from satellite cells, lineage tracing has identified a variety of stem cell populations in skeletal muscle and the satellite cell compartment including side population cells (Challen et al., 2006), mesenchymal stromal cells (Boppart et al., 2013), pericytes (Dellavalle et al., 2007), interstitial stem cells (Cottle et al., 2017), and fibro/adipogenic progenitors (FAPs) (Joe et al., 2010). These cell populations proliferate following muscle injury and have many features in common, namely cell-surface marker expression and multipotency. *In vivo* transplantation assays and *in vitro* co-culture experiments demonstrated that these endogenous stem cell types play an important role in promoting self-renewal, proliferation, and commitment to myogenic differentiation of satellite cells (Peault et al., 2007; Dunn et al., 2019).

Satellite cells are best described by the expression of their canonical marker, Pax7. Several studies have demonstrated that Pax7 expression is necessary for the normal function of satellite cells during both neonatal and adult skeletal muscle myogenesis. By utilizing genetic models of the conditional deletion of Pax7^+^ cells using tamoxifen-inducible diphtheria toxin from the *Rosa* locus, studies showed that skeletal muscle regeneration was impeded following muscle injury (Lepper et al., 2011; Murphy et al., 2011; Sambasivan et al., 2011). Interestingly, skeletal muscle without satellite cells was incapable of regeneration even after transplantation of the Pax7-deficient muscle into a healthy host muscle (Lepper et al., 2011; Sambasivan et al., 2011). Furthermore, observations by our group also indicated that deletion of Pax7 in adult satellite cells, by using floxed alleles and tamoxifen-induced inactivation, resulted in a significant reduction in regeneration as evidenced by reduced formation of myofibers (von Maltzahn et al., 2013). Notably, other stem cell populations within the skeletal muscle did not compensate for the loss of Pax7^+^ satellite cells, and the satellite cell depletion and regeneration defect could only be rescued upon transplantation of Pax7^+^ cells. Together, these findings provide compelling evidence that these stem cell populations can only contribute to muscle repair in the satellite cell rich environment during injury-induced muscle regeneration, and these observations emphasize the important implication of satellite cells for cell-based transplantation assays.

## Transplantation-based approach to assess satellite cell functions

Numerous studies have employed transplantation strategies to assess the self-renewal and differentiation potential of satellite cells. In an early study, satellite cells-expressing GFP were directly isolated from adult muscle tissues and engrafted into the dystrophin-deficient *mdx* nude muscle and were found to contribute to both myofiber repair and to the muscle satellite cell reservoir (Montarras et al., 2005). Recently, in a two-armed transplantation assay, Kyba and colleagues (Arpke et al., 2021), transplanted simultaneously donor-derived Pax7-ZsGreen satellite cells into both *tibialis anterior* (TA) muscles of young and old NSG-*mdx* mice. One limb was used for flow cytometry to quantify the number of undifferentiated (ZsGreen^+^) satellite cells 1 month after transplantation, i.e., measuring self-renewal capability. The other limb was used in histological analysis to determine the number of dystophin^+^ fibers, quantifying the contribution of the donor cells to form new myofiber. They found that the ability of satellite cells to self-renew and to properly differentiate was comparable between the old and young mice. In another study, Pax7-YFP knock in satellite cells were isolated by FACS and transplanted into injury-induced regenerating limb muscle of *mdx* mice. It was found that YFP^+^ donor-derived satellite cells were able to repopulate the satellite cell compartment, suggesting that this population is capable of self-renewal. They, also observed that could also differentiate and restore dystrophin in *mdx* myofibers (Kitajima and Ono, 2018).

Most importantly, transplantation experiments from our group using the *Myf5-Cre*/*ROSA-YFP* lineage tracing technique, demonstrated satellite cell heterogeneity through their stemness and indicated that about 10% of satellite cells are true stem cells (Kuang et al., 2007). Furthermore, we observed that only the transplanted YFP^−^ satellite cells could produce both self-renewed Pax7^+^/YFP^−^ satellite stem cells and Pax7^+^/YFP^+^ satellite committed progenitor cells through apical-basal asymmetric division. By contrast, isolation and transplantation of YFP^+^ satellite cells resulted in poor survival and migration, lower engraftment capacity, and precociously differentiated. Similarly, using transgenic *Pax7-nGFP* reporter mice, Rocheteau et al. 2012a) revealed that a *Pax7* ^high^ cell population is less primed to commitment and maintain stemness in quiescent satellite cells. They showed that proliferating satellite cells with high Pax7 expression asymmetrically segregate their DNA during cell division, while the Pax7^low^ cell population distributes their DNA randomly. Moreover, by serial transplantation they demonstrated that the Pax7 high population has a higher engraftment potential, can self-renew, and give rise to committed progenitors, while the Pax7 low population has a higher propensity for differentiation.

A study by Blau’s group used cytometry by time-of-flight (CyTOF) to identify novel populations of MuSCs based on their surface markers. They discovered two progenitor cell populations, P1 and P2, expressing either CD9 alone or both CD9 and CD104, respectively. Notably, the CD9^+^/CD104^−^ subpopulation demonstrated higher regenerative capacity upon transplantation into irradiated muscle, suggesting that they possess a greater self-renewal potential to replenish the satellite cell compartment (Porpiglia et al., 2017).

Accumulating evidence demonstrates that primary cilia are critical for satellite cell maintenance in a quiescent state. During quiescence, primary cilia are shown on MuSCc surfaces but rapidly disassembles upon activation and reassembles preferentially in self-renewing MuSCs (Jaafar Marican et al., 2016). Adult MuSCs devoid of primary cilia exhibit a defect in muscle regeneration, poor engraftment, and an increased cell cycle transcriptome signature. The histone deacetylase 6 (Hdac6) has been shown to promote the disassembly of the primary cilium (Palla et al., 2020). *In vitro* culture experiments demonstrated that tubastatin A (TubA), an agonist of Hdac6, inhibits primary cilium resorption, thereby maintaining satellite cells in their quiescent state and increasing their survival rate. Moreover, treatment of MuSCs with TubA improved engraftment potential upon transplantation (Arjona et al., 2022). In addition, data from our group demonstrated that primary cilia-mediated regulation of GLI3 transcription factor plays an important role in keeping the satellite cells in their dormant G0 state and inhibiting their activation (Brun et al., 2022).

Interestingly, our group has identified that satellite cells also represent heterogeneity with regard to their cell fate capacity (Yin et al., 2013). By clonal analysis, we identified that a subset of satellite cells is bi-potential, thereby being capable of generating myogenic progenitors, or alternatively, brown adipocytes. The switch from myogenic to brown adipogenic fates is regulated by microRNA-133 (miR-133). Downregulation of miR-133 results in upregulation of Prdm16, a brown adipose determination factor. Both muscle and brown adipose tissue (BAT) increase metabolic rates by regulating thermogenesis. Brown adipose is relatively rare in adult humans, and thus transplantation of BAT or promoting endogenous production of BAT from satellite stem cells in muscle could provide a novel approach to stimulate BAT volume, regulate energy expenditure, and impair the development of obesity.

**FIGURE 1:**
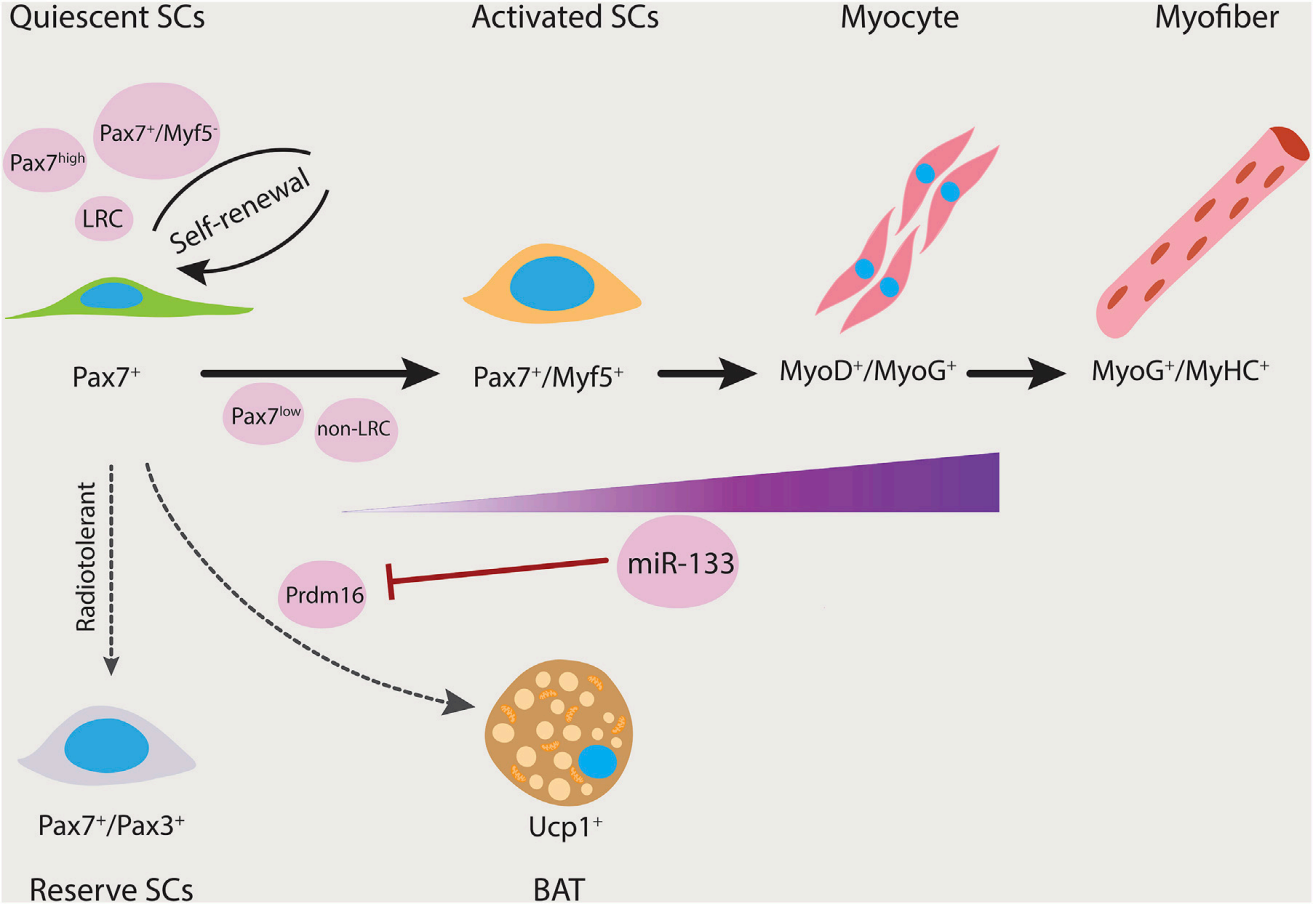
Satellite cell heterogeneity. Satellite cells can give rise to both myogenic and brown adipogenic lineages. miR-133 expression is upregulated as the myogenic program progresses. Prdm16 regulates satellite cell differentiation into brown adipocytes and its expression is inhibited by miR-133. Thereby, miR-133 controls brown adipose determination (dashed arrow). Subsets of satellite cells have differing functional potential (black arrows). Pax7^+^/Myf5^−^, Pax7^high^, and long-term label-retaining populations have the ability to repopulate the stem cell pool. Furthermore, Pax7^+^/Pax3^+^ is a rare subpopulation that is resistant to radiation and contains reserve stem cell properties. LRC, label-retaining cells; non-LRC, non-label-retaining cells; SCs, satellite cells.

Altogether, multiple lines of evidence utilized transplantation experiments to demonstrate the heterogeneity of satellite cells and that satellite cells vary in their stemness and lineage commitment (Figure 1). However, the stemness properties between satellite stem cell subpopulations (e.g., Pax7^high^, Pax7^+^/YFP^−^, and non-LRCs) or the differentiation potential between satellite myogenic cell subpopulations (e.g., Pax7^low^, Pax7^+^/YFP^+^, and non-LRCs) are currently not known. With the emergence of single-cell technologies, one can delineate the differences between these subpopulations based on their gene expression and protein signatures, lineage commitment, and potentially reveal novel cell populations. It will also be interesting to explore the role of the subpopulations of satellite cells in fiber types specification. The lineage tracing mouse models would allow for flow cytometric purification of the satellite cell subtypes from any muscles. Transplanting these cell subtypes into a regenerating host muscle, then harvesting in 1 month, and immunostaining for terminally differentiated Myosin Heavy Chains (MyHC) would allow for the visualization of different muscle fiber types. This experiment would then determine the satellite cell subpopulations contribution to specific fiber types.

## Irradiation and injury-preconditioning the host environment for efficient transplantation

A number of studies have focused on approaches to generate a supportive environment within the skeletal muscle to enhance the engraftment efficiency of the transplanted cells. High dose irradiation of the host mouse muscle has been shown to be an effective preconditioning procedure for satellite stem cell transplantation (Morgan et al., 2002; Boldrin et al., 2012). Previous work has suggested that irradiation results in a substantial proliferation of the transplanted cells and their progeny (Beauchamp et al., 1999). It also promotes the transplanted cells to form high numbers of myofibers, and migration to neighbouring muscles is enhanced.

Subjecting *mdx* nude mice muscles to an 18 Gy dose of irradiation dramatically depletes the satellite cell pool while keeping the basal lamina intact and promoting cell engraftment. However, when the mice were subjected to a 25 Gy dose of irradiation, complete ablation of the host satellite cells resulted, thus impeding donor cell engraftment (Boldrin et al., 2012). This suggests in order to keep the host environment functional, it requires some viable host satellite cells to allow robust donor-derived engraftment to occur. While high dose irradiation is not feasible in a clinical setting, characterizing the pertaining molecular mechanisms could provide more clinically-appropriate strategies to alter the host muscle environment and ameliorate donor cell engraftment. Recent work from Morgan’s group demonstrated that the innate immune response genes were upregulated 3 days after irradiation compared to non-irradiated muscles. Mice with defective innate immune response showed significantly less donor-derived cell engraftment compared to the control. Therefore, this study suggests that irradiation-mediated innate immune system activation plays an important role in increasing donor satellite cell engraftment. It would be of interest to elucidate what cell types are involved within the irradiated host muscle and the mechanism(s) by which they promote donor cell engraftment (Doreste et al., 2020).

In addition to irradiation, muscle pre-treatment regimes to induce injury such as mechanical injury (cryoinjury) (Le et al., 2016), injection of myotoxins (cardiotoxin and notexin) (Heslop et al., 2000; Garry et al., 2016), or chemical agent (BaCl_2_) (Tierney and Sacco, 2016) are commonly used to augment engraftment. Myotoxins and BaCl_2_ injure skeletal muscle by compound mechanisms that result in selective breakdown of endogenous myofiber membranes without affecting the blood vessels, nerves, or muscle fiber basal lamina (Couteaux et al., 1988; Harris, 2003). Notably, cryoinjury is the most damaging model resulting in depletion of satellite cells up to 96% and in the destruction of their environment, forming a “dead zone” lacking viable cells (Hardy et al., 2016).

A study published by Hardy and colleagues compared these different commonly used injury models in immune-competent mice (Hardy et al., 2016). It was found that all these injury models display similar necrosis at onset, and that a full regeneration was achieved 1 month after injury. They, however, demonstrated significant variation in parameters such as satellite cell survival and expansion, re-vascularization of the tissue, and immune profile during regeneration. Thereby each model represents a distinct regeneration profile. Interestingly, the production of the inflammatory cytokines in the cardiotoxin injury model was restored to normal levels once histological analysis of the muscle tissue revealed a complete regeneration. However, in the other models, despite a normal histological appearance, the expression level of cytokines was never returned to normal, suggesting prolonged inflammation. In another report (Silva-Barbosa et al., 2005), however, the level of inflammatory cell infiltration was higher in the host TA muscles of the cardiotoxin-treated immunocompromised mice when compared to that induced by cryoinjury. This study also found more transplanted donor cells after cryoinjury when compared to the cardiotoxin group. The authors thus claimed that the difference observed between the two injury models, can be at least partially, explained by the higher number of inflammatory cells following cardiotoxin-induced injury.

It has been demonstrated that the interaction of the satellite cell with inflammatory cells (mostly the macrophages) is crucial for efficient regeneration (Chazaud et al., 2003; Yang and Hu, 2018). In addition, most studies focusing on muscle injury models have been utilizing immune-competent mice, who display varying responses. Thus, it would be interesting to investigate the effect of these injury methods in immunodeficient mice, where inflammation may be suppressed. Taken together, while the muscle tissue is able to fully regenerate in all these injury models, one should consider that the alterations in the satellite cells microenvironment (niche) and the trajectories of the regenerative process vary considerably among these injury models. Thus, the preferred type of injury model depends on the study design and desired outcome.

Additionally, Olwin and his group (Hall et al., 2010) demonstrated that transplantation combined with an injury (Cardiotoxin or BaCl_2_) modifies the host environment and results in a greater regenerative capacity in the engrafted satellite cells, thus eliciting an increase in muscle mass and muscle function. Similar observations have been made by our group (Price et al., 2014; Wang et al., 2019; Feige and Rudnicki, 2020) whereby the skeletal muscle transplantation experiments incorporated cardiotoxin injection of the host muscle prior to the transplantation, resulted in efficient engraftment into immunocompromised *mdx* recipient mice.

To further investigate the effect of the modulation of the host muscle environment on skeletal muscle stem cell regenerative potential, Morgan and colleagues (Meng et al., 2015) used human skeletal muscle-derived stem cell types in two immunodeficient mouse strains (*mdx* nude and C5^−^/γ chain^−^/Rag2^−^) that had been subjected to either irradiation, or cryoinjury, or both. Cryoinjury or a combination of cryoinjury and irradiation resulted in higher muscle fibers of donor origin in both mouse strains, than by irradiation alone. An interesting finding was that when the two mouse strains were challenged with similar conditions, the C5^−^/γ chain^−^/Rag2^−^ mice was shown to be a superior recipient mouse strain than *mdx* nude mice for transplantation of human muscle stem cells, mostly due to the fact that the C5^−^/γ chain^−^/Rag2 mice have a more profound immunodeficiency. This study revealed that the ability of the exogenous stem cells to contribute to muscle regeneration is affected by the different environments present in the recipient mouse strains and also is dependent on the types of modulations of the host muscle prior to cell transplantation (Meng et al., 2015).

**FIGURE 2:**
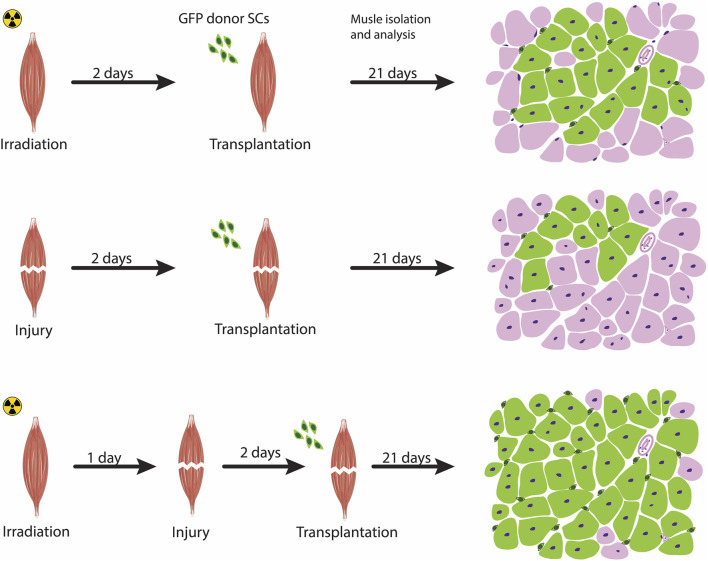
Preconditioning the host muscle environment prior to transplantation. Pre-treatment strategies to modulate the host muscle environment includes irradiation (top panel), injury-induced (middle panel), or a combination of irradiation and acute injury (bottom panel). Acute muscle injury combined with irradiation exhibit a high level of engraftment, as schematized by a transverse section of a transplanted muscle showing an increased contribution of fibers by transplanted cells (marked as green fibers) compared with the other two approaches. The Green cells surrounding the fibers represent satellite cells donor-derived cells that have undergone self-renewal.

Subsequently, the benefit of irradiation coupled with injury was further confirmed in transplantation assays of low numbers of hindlimb satellite cells (as few as 300 cells) with and without irradiation and showed that the contribution of satellite cells to repopulate the niche and form new myofibers were substantially reduced in the absence of irradiation (Arpke et al., 2021). Data from the irradiation-tolerant immunodeficient NOD-Rag (NRG) mice (Pearson et al., 2008) undergoing a high level of irradiation at 25 Gy in combination with cardiotoxin injection showed that this preconditioning of the host environment not only eliminated almost all of the host tissue of the mouse origin but also the development of hybrid human donor-murine host fibers (Sakellariou et al., 2016). Taken together, these observations support the notion that acute muscle injury in combination with irradiation as a strategy to induce changes in the cellular composition of the host environment prior to transplantation results in an extensive self-renewal to replenish the satellite cell pool and contribution to muscle repair (Figure 2).

## Assessing the success of engraftment

Optimizing stem cell-based transplantation assays requires a good understanding of their cellular kinetics, differentiation potential, and fate following engraftment. Therefore, reliable imaging assays play a critical role in the assessment of engraftment. The classical approach for evaluating engraftment in animals is the analysis of muscle histopathology. Donor-derived cells are typically genetically labeled with reporter genes encoding for the *GFP* gene, β-galactosidase gene (Asakura and Rudnicki, 2002; Asakura et al., 2007), or the alkaline phosphatase gene (Gerard et al., 2009). These reporter genes are typically introduced into cells using transfection or viral infection assays. Alternatively, the cells are derived from transgenic reporter mice carrying a transgene.

For example, primary myoblasts expressing tdTomato derived from transgenic *Pax7-CreER;R26R-tdTomato* mice after treatment with tamoxifen were transplanted into an immunocompromised host. Scoring for tdTomato + myofibers that represents fusion of the transplanted cells was performed 1 week following transplantation to provide insights into the behavior of the transplanted tdTomato + myoblasts (Bentzinger et al., 2014). In another example from our group, the TA muscle of immunocompromised *mdx* mice was pre-injured with cardiotoxin and transplanted with Pax7-ZsGreen expressing satellite cells freshly sorted from reporter mice (Price et al., 2014). The benefit of using *mdx* mouse model is that the restoration of dystrophin by transplanted donor stem cells can be assessed. To evaluate the efficacy of satellite cell transplantations in mice, Chakkalakal et al. (2014) used a novel lineage tracing system, the Tet-on-H2B-GFP transgenic reporter mice. H2B is a histone protein that plays a role in the DNA packaging of eukaryotic cells, thereby any H2B-GFP signal will be restricted to nucleus. This is crucial as the GFP signal will not diffuse through the muscle fibers once the H2B-GFP^+^ cells have fused into the fibers. In addition, recent studies using endogenous reporter *Myf5-Cre/R26R-nTnG* mouse model allowed for discrimination between the committed satellite myogenic cells (nGFP^Pos^) from satellite stem cells (nTdT^Pos^) (Wang et al., 2019; Feige and Rudnicki, 2020).

The conventional histological analysis is, however, extremely challenging to quantify, time-consuming, and dependent on sacrificing the experimental animals at different time points. Thus, over the past years, investigators sought non-invasive or minimally invasive methods. The Blau laboratory (Sacco et al., 2008) discovered a non-invasive *in vivo* bioluminescence imaging (BLI) technique to monitor muscle stem cells behaviour by crossing *Myf5-nLacZHet* mice with firefly luciferase (FLuc) transgenic mice. The BLI assay allows the dynamics of stem cell behavior to be evaluated in ways not feasible when using conventional histological approaches. For example, viability, proliferation, and engraftment of the donor-derived muscle stem cells can be monitored following transplantation until muscle homeostasis is reached. Additionally, the response of the stem cells to injury and their contribution to regeneration can be tracked over time without the need to sacrifice animals at different time point. Of note, the bioluminescence signal penetration depth is maximal (upto 2 cm) in the skin and muscle tissues and thus reduction in signal is not a limiting factor (Massoud and Gambhir, 2003). Interestingly, reports from Rando’s group (Maguire et al., 2013; Filareto et al., 2018) described “regeneration and degeneration reporter” mice strains by conditionally expressing luciferase in satellite cells or in the myofiber of the skeletal muscle, respectively. Mating these mice with dystrophic mouse models, such as *mdx*, allowed for the assessment of disease progression directly in living animals overtime. These results collectively highlight the usefulness of luciferase reporter animals as quantitative and robust tools for the non-invasive long-term assessment of experimental therapeutic interventions for animal models of muscular dystrophy. We note that although the histological analysis is technically challenging and time-consuming, it still offers certain advantages over BLI, in that it allows for tracing of multi-lineage commitment of satellite cell subtypes and exploring satellite cell heterogeneity.

## Approaches to overcome transplantation challenges

While satellite cells represent the ideal cell type to be utilized in transplantation assays not only because of their remarkable regenerative capacity but also their contribution to the satellite cell pool, a number of caveats limit their engraftment potential. Satellite cells’ remarkable regenerative capacity is rapidly lost once they are isolated and expanded in culture. For instance, the engraftment ability of the mouse myoblasts grown *ex vivo* after 3 days in culture was significantly reduced (Montarras et al., 2005; Sacco et al., 2008). Similarly, growing canine primary myoblasts *in vitro* cultures demonstrated lower engraftment capacity compared with freshly isolated canine muscle stem cells (Parker et al., 2012). Expansion of human myoblasts *in vitro* prior to transplantation into DMD patients also resulted in a considerable reduction in engraftment efficiency and failure to contribute to the muscle stem cell reservoir (Gussoni et al., 1992; Bouchentouf et al., 2007). Therefore, a number of recent studies have been focusing in developing approaches to alleviate this problem to a certain extent. Remarkably, manipulation of biophysical properties, in terms of tissue stiffness, geometry, and extracellular matrix composition, helped researchers to develop culture systems to mimic the natural microenvironment of the satellite stem cell niche. In contrast to rigid plastic cell culture dishes, growing the satellite stem cells on soft hydrogels, which mimic the plastic module of muscle tissue, supports *in vitro* self-renewal and thus can help maintain engraftment and niche repopulation capacity (Gilbert et al., 2010). Moreover, bioengineering strategies have also been employed to enhance the delivery, survival, and maturation of transplanted cells following expansion. Generation of injectable, encapsulating 3D biomaterials, such as synthetic macromers (Han et al., 2018) and bioactive hydrogels (Quarta et al., 2017), that mimic the hierarchical structural organization of muscle and can be used to deliver growth factors has been shown to boost transplantation potential in *mdx* muscles.

Alternative approaches rely on supplementation of culture media with small molecules to modulate signaling pathways that aim to retain stemness properties while inhibiting terminal myogenic commitment. Work from our own lab has identified the Wnt7a/Fzd7/Vangl2 and EGFR/Aurka pathways as key modulators of the symmetric and asymmetric satellite stem cell divisions, respectively (Le Grand et al., 2009; Wang et al., 2019). Wnt7a and EGFR control satellite stem cell division in the myofiber niche by regulating centrosome recruitment. Wnt7a was shown to increase the pool of self-renewing stem cells by favoring symmetric division, while EGFR promotes asymmetric division and thus increases the pool of committed progenitors. EGF treatment *in vivo* rescued asymmetric divisions in dystrophin-deficient satellite stem cells, thereby stimulating the productive generation of myogenic progenitors and enhancing regeneration and muscle strength (Wang et al., 2019).

The ability of satellite cells to regenerate declines with age largely due to the decrease in their number and molecular changes in their niche. In aged animals, the Notch-p53 axis is downregulated, resulting in mitotic cell death and thus impaired muscle repair (Liu et al., 2018). In contrast, some key morphogenic signaling pathways, such as Wnt (Brack et al., 2007), JAK-STAT (Price et al., 2014), and TGFβ/pSmad3 (Carlson et al., 2008) are prematurely activated in aged muscle, causing inflammation and a defect in satellite cell proliferation. The application of heterochronic parabiosis strategies restored a more “youthful” calibration of these pathways in the aged satellite cells, and consequently promoted rejuvenation of the aged tissue and significant muscle regeneration (Conboy and Rando, 2012; Conboy et al., 2013). In addition, pharmacological targeting of the aged satellite cells to enhance or attenuate these signaling pathways in defined culture conditions, prior to their transplantation into old animals, contributed to new myofiber formation with enhanced efficiency than the control untreated satellite cells (Price et al., 2014; Liu et al., 2018; Mehdipour et al., 2019).

The limited migratory capacity of transplanted cells is another substantial issue associated with poor cell engraftment. Several strategies have been explored to enhance satellite cell migration upon intramuscular transplantation in mice, most of which involve the activation of specific signaling pathways. We showed that Wnt7a regulated the motility of the satellite cells through activation of noncanonical Wnt signaling, thereby improving donor cell engraftment ability and increasing muscle strength upon transplantation into dystrophic muscles (Bentzinger et al., 2014). Coinjection of mouse pro-inflammatory macrophages along with satellite cells or myoblasts showed improved donor-derived regeneration, which was attributed to improved donor cell proliferation, migration, and delayed differentiation (Lesault et al., 2012; Rybalko et al., 2015). miR-708 is a quiescence-specific mirtron that acts as a downstream target of Notch signaling to repress Tensin3 and thus maintain satellite cells within their quiescent niche. Tensin3 is a major component of focal adhesion (FA)-associated proteins that play a critical role in regulating cell adhesion and migration. Therefore, Notch signaling maintains satellite cell quiescence by antagonizing the migratory machinery. It thus remains interesting to further investigate whether downregulation of Notch signaling and hence miR-708 would result in increased migration capacity of the satellite cells and thereby enhanced engraftment efficiency (Baghdadi et al., 2018).

## Concluding remarks and perspectives

Satellite cells are the critical cellular source for muscle regeneration, and hence they are the most promising means in cell engraftment assays. As discussed in this review, they possess self-renewal and myogenic differentiation potential, and can be easily identified based on their location and molecular signature. Moreover, growing evidence indicates that the satellite cell population is not homogenous and that the transplantation approaches involving them provide an important tool to understand their fate decisions and heterogeneity *in vivo*. Additionally, studies using single-cell technologies in combination with transplantation approaches can represent promising avenues to further investigate the heterogeneity of the satellite cell compartment. This can help identify novel subpopulations and delineate a myogenic trajectory from quiescent stem cells to committed progenitors *in vivo* in skeletal muscle. Therefore, such studies will be valuable to characterize satellite cell regulation to gain a deeper understanding of the components of homeostasis and the regeneration capabilities of skeletal muscle.

Significant progress has been made in recent years in the isolation of satellite cells, leading to the extensive characterization of human satellite cells and their transplantation (Garcia et al., 2017). The transplantation of human satellite cells into mice demonstrated that these cells can successfully integrate with irradiated host muscles to produce muscle fibers and self-renew (Marg et al., 2014). The ability to grow human muscle fibers in a host animal can be helpful for many research applications, including the study of their cellular heterogeneity and the potential development and testing of therapeutics in preclinical models.
